# Relationships between Motor and Executive Functions and the Effect of an Acute Coordinative Intervention on Executive Functions in Kindergartners

**DOI:** 10.3389/fpsyg.2017.00859

**Published:** 2017-05-30

**Authors:** Marion Stein, Max Auerswald, Mirjam Ebersbach

**Affiliations:** ^1^Department of Psychology, University of KasselKassel, Germany; ^2^Department of Quantitative Methods in Psychology, Ulm UniversityUlm, Germany

**Keywords:** executive functions, physical activity, acute exercise, coordinative intervention, kindergartners, cognition

## Abstract

There is growing evidence indicating positive, causal effects of acute physical activity on cognitive performance of school children, adolescents, and adults. However, only a few studies examined these effects in kindergartners, even though correlational studies suggest moderate relationships between motor and cognitive functions in this age group. One aim of the present study was to examine the correlational relationships between motor and executive functions among 5- to 6-year-olds. Another aim was to test whether an acute coordinative intervention, which was adapted to the individual motor functions of the children, causally affected different executive functions (i.e., motor inhibition, cognitive inhibition, and shifting). Kindergartners (*N* = 102) were randomly assigned either to a coordinative intervention (20 min) or to a control condition (20 min). The coordination group performed five bimanual exercises (e.g., throwing/kicking balls onto targets with the right and left hand/foot), whereas the control group took part in five simple activities that hardly involved coordination skills (e.g., stamping). Children’s motor functions were assessed with the Movement Assessment Battery for Children 2 (Petermann, 2009) in a pre-test (T1), 1 week before the intervention took place. Motor inhibition was assessed with the Simon says task (Carlson and Wang, 2007), inhibition and shifting were assessed with the Hearts and Flowers task (Davidson et al., 2006) in the pre-test and again in a post-test (T2) immediately after the interventions. Results revealed significant correlations between motor functions and executive functions (especially shifting) at T1. There was no overall effect of the intervention. However, explorative analyses indicated a three-way interaction, with the intervention leading to accuracy gains only in the motor inhibition task and only if it was tested directly after the intervention. As an unexpected effect, this result needs to be treated with caution but may indicate that the effect of acute coordinative exercise is temporally limited and emerges only for motor inhibition, but not for cognitive inhibition or shifting. More generally, in contrast to other studies including older participants and endurance exercises, no general effect of an acute coordinative intervention on executive functions was revealed for kindergartners.

## Introduction

Children’s increasing use of technological devices, such as smartphones or computers, promotes a sedentary lifestyle at least in industrial societies. The minimum of 60 min of daily physical activity, as recommended by the World Health Organization [WHO] (2010), is accomplished only by one third of the children in Germany (Manz et al., 2014) and in the United States (Centers for Disease Control and Prevention, 2008). This has not only an effect on their physical development and health (Janssen and LeBlanc, 2010) but may also affect their cognitive development. Correlative studies with children revealed positive relationships between cognitive functions and physical activity (Campbell et al., 2002; Becker et al., 2014) as well as between cognitive and motor functions (Livesey et al., 2006; Davis et al., 2011). Moreover, intervention studies suggested that both acute (i.e., one-time) and chronic (i.e., repeated) physical exercise may cause beneficial effects on subsequent cognitive functions of children aged older than 6 years, adolescents, and adults (for a meta-analysis, see Sibley and Etnier, 2003; Verburgh et al., 2014).

These positive effects of physical activity on cognitive functions can be explained by physiological and developmental mechanisms: First, physical activity might elicit physiological changes, such as enhancing the cerebral blood flow (e.g., Herholz et al., 1987) and increasing the release of neurotransmitters – factors that are assumed to positively affect cognitive functions (Chmura et al., 1998; Winter et al., 2007). Second, motor development and cognitive development are closely interrelated (e.g., Sibley and Etnier, 2003). According to Piaget (1972), the first concepts that are acquired in infancy are based on sensorimotor experiences. The skills and relations learned through these experiences can be transferred to cognitive problems and therewith form the basis of further cognitive development. In addition, acquiring and executing new and more complex motor movements requires and stimulates cognitive functions (Ackerman, 1987; Best, 2010). This stimulation occurs, for instance, also during team sports (e.g., soccer), where players have to cooperate with team mates, anticipate their movements, develop strategies, and switch between changing task conditions (Best, 2010). The interrelation between motor and cognitive development is also reflected by the existence of neuronal connections between the cerebellum (responsible, for instance, for the control and temporal coordination of movements) and the prefrontal cortex (responsible, for instance, for executive functions; Raichle et al., 1994; Diamond, 2000). The simultaneous activation of the cerebellum and prefrontal cortex primarily occurs in cognitive or motor tasks, which are complex, unknown, require fast reactions, or underlie changing conditions (Diamond, 2000). Thus, it would be suggestive to use the relationship between motor and cognitive functions to enhance one or the other by means of interventions.

Besides benefitting from physical activity in the long run, it could also be reasonable that an acute bout of physical activity induces a short-term increase of cognitive performance, for example, if it is executed before a school test or a difficult learning situation. Most of the intervention studies reporting positive effects of acute physical activity on cognitive functions of children used *aerobic exercise interventions* and were conducted in individual settings, in which the physical intensity of the intervention was strictly controlled (e.g., Hillman et al., 2009; Ellemberg and St-Louis-Deschênes, 2010; Pontifex et al., 2013). Only a few studies examined the efficacy of acute *coordinative interventions* on cognitive functions so far. These acute coordinative interventions were often conducted in group settings and were therewith less controlled. Nevertheless, they also yielded positive effects on cognitive functions (e.g., Budde et al., 2008; Jäger et al., 2014). Despite the small number of studies including acute coordinative interventions, several researchers theoretically assumed a superior effect of coordinative in comparison to aerobic exercise interventions (e.g., Budde et al., 2008; Best, 2010). This assumption is grounded in higher demands of motor control and cognitive functions (e.g., spatial orientation) for coordinative exercises compared to aerobic exercises (Budde et al., 2008; Voelcker-Rehage et al., 2011). Coordinative interventions might thus not only evoke physiological changes, as already mentioned (e.g., general enhanced release of neurotransmitters in the brain), but additionally stimulate the neuronal network between cerebellum and prefrontal cortex due to their higher motor and cognitive complexity. This stimulation could function as a pre-activation for the subsequent cognitive performance (Diamond, 2000; Budde et al., 2008), for instance, by an increased release of neurotransmitters in these specific areas.

However, the question of how complex or how demanding a coordinative exercise actually is for a child depends mainly on the level of his or her motor and cognitive functions (McMorris, 2009). Due to this interaction between interventional and individual factors, the abilities of each child should be taken into account when designing a coordinative intervention, which was rarely done so far. In the current study, we aimed at enhancing the executive functions of kindergartners (i.e., 5- to 6-year-olds) by an individually executed, acute coordinative intervention that was adapted to the kindergartners’ individual motor performance.

In particular, executive functions can be positively affected by both kinds of physical activity (e.g., Tomporowski et al., 2008b; McMorris and Hale, 2012). Executive functions are fundamental cognitive processes, which are responsible for goal-directed behavior, especially in new and not automated situations (Banich, 2009). They include updating, inhibition, and shifting. *Updating* means to monitor and modify mental representations in the working memory. This is required, for instance, in order to remember plans and to evaluate available behavioral alternatives. *Inhibition* involves the suppression of predominant and automated reactions as well as being resistant against distraction. It includes controlling one’s behavior and attention, instead of being affected by external stimuli and emotions. *Shifting* allows to switch attention between different tasks or rules, enabling fast and flexible adjustments to changing conditions (Miyake et al., 2000; Diamond, 2006). Executive functions play a central role in current and future academic achievement (St Clair-Thompson and Gathercole, 2006; Bull et al., 2008; Best et al., 2011) as well as in social competence and the occurrence of externalized behavior (Nigg et al., 1999; Ciairano et al., 2007; Best et al., 2009). Therewith, early interventions that enhance children’s executive functions before they enter school might be helpful to promote their social and academic development.

Until now, only a few studies examined the effect of an acute bout of physical activity on cognitive functions of kindergartners (Palmer et al., 2013; Mierau et al., 2014), which is astonishing given the fact that this age phase can be conceived as a sensitive period, in which cognitive and brain development rapidly progress (Brown and Jernigan, 2012). In particular, inhibition as one of the executive functions develops markedly at kindergarten age (Best et al., 2009; Best and Miller, 2010; Röthlisberger et al., 2010). In general, a positive effect of acute physical activity on inhibition has already been demonstrated for different age groups including older children (e.g., Hillman et al., 2009; Ellemberg and St-Louis-Deschênes, 2010; for a review see Barenberg et al., 2011). However, the only study with kindergartners in this regard, examining the effect of an acute coordinative group intervention (Palmer et al., 2013), showed only a marginal effect on inhibition. Furthermore, the study of Mierau et al. (2014) that included an acute group intervention based on aerobic exercise games (e.g., soccer), failed to find an effect on shifting of kindergartners. Therefore, further studies are needed to clarify whether effects of acute physical activity interventions, revealed for older children, adolescents, and adults, emerge in kindergartners, too.

The present study had two aims: First, the correlational relationships between motor and executive functions in kindergartners were examined. Several studies reported a positive, moderate relationship between these functions in this age group (Livesey et al., 2006; Cameron et al., 2012; Roebers et al., 2014). However, only a few studies included shifting (e.g., Mierau et al., 2016) and motor inhibition (Sereno et al., 2006) as aspects of executive functions. Motor inhibition requires suppressing a dominant motor action, while cognitive inhibition requires focusing the attention to a relevant cue and ignoring an irrelevant cue (Sereno et al., 2006). To consider a broader variety of executive functions, *shifting* and *motor inhibition* were included in addition to *cognitive inhibition* in the current study.

Second, we investigated whether an acute, adaptive coordinative intervention yielded causal effects on specific executive functions of kindergartners. Besides an expected, general effect of the intervention, we assumed that the three assessed executive functions (i.e., motor inhibition, cognitive inhibition, shifting) would be affected differently. The efficacy of an acute physical activity intervention on cognitive inhibition of older children and adolescents could be shown in several studies (e.g., Jäger et al., 2014; for a review see Verburgh et al., 2014). However, it is still unclear if this finding could be replicated in kindergartners. Furthermore, some researchers assumed that the efficacy of physical activity on executive functions depends on the developmental status of the child and of the executive function, in that higher developed executive functions should benefit more (Tomporowski et al., 2008b; Best, 2010). Besides the activation of common brain regions in the prefrontal cortex, different executive functions are also associated with distinct brain regions, which follow other developmental courses (Olson and Luciana, 2008; Best and Miller, 2010). In particular, brain regions associated with shifting fully mature only between late adolescence and early adulthood (Olson and Luciana, 2008). Accordingly, the neurophysiological basis for shifting could be too premature among kindergartners to show great changes due to physical activity in this age group. This led us to the assumption that cognitive inhibition, which is better developed than shifting in kindergartners, therefore should benefit more than shifting from physical activity. Furthermore, the efficacy of an acute bout of physical activity on motor inhibition as one aspect of executive functions was rarely examined. We expected that the coordinative intervention would be more effective for motor inhibition than for cognitive inhibition or shifting due to the greater congruency between the coordinative intervention and the motor inhibition tasks: Both require that whole-body movements are inhibited.

## Materials and Methods

### Design

The experiment followed a 2 × 2 × 3 mixed design with experimental condition (i.e., acute coordinative intervention condition vs. control condition) and order of the tasks assessing executive functions (i.e., the “Hearts-and-Flowers” task to assess cognitive inhibition and shifting first or the “Simon-says” task to assess motor inhibition first) as between-subjects factors and type of executive function (motor inhibition vs. cognitive inhibition vs. shifting) as within-subjects factor. Accuracy and reaction times in the executive function tasks, measured 1 week before the experimental conditions (T1), were included as predictors in the respective linear mixed model. The dependent variables were accuracy and mean reaction times in the executive function tasks, conducted immediately after the experimental conditions (T2). During the coordinative intervention, motor performance (e.g., how often a ball was thrown at a target and how often the target was hit) was recorded and physical intensity of the intervention was assessed in both conditions by recording children’s heart rates. In addition, children’s motor functions were assessed in T1 to test whether they yielded correlations with executive functions at T1.

### Sample

Ethical consent for the experiment was obtained from the faculty’s ethic committee^1^. Initially, 135 kindergartners were recruited from nine local kindergartens in a medium-sized town in Germany after their parents signed a consent form. The children had intermediate socio-economic backgrounds and spoke and comprehended German fluently. Several children had to be excluded due to being absent on the day of the experimental intervention (*n* = 13), failures in measuring – or missing – the targeted physical intensity level in the coordinative intervention (*n* = 11) or lacking motivation or comprehension during the executive function tasks (*n* = 11). The remaining sample consisted of 101 kindergartners aged 60 to 85 months. These children were randomly assigned to one of two experimental conditions: an acute coordinative intervention condition (*n* = 48, mean age: *M* = 72.2 months, *SD* = 5.2, 24 males) or a control condition (*n* = 53, mean age: *M* = 72.3 months, *SD* = 6.9, 25 males). Preliminary analyses revealed that there were no significant differences between the drop-outs and the remaining sample with regard to the motor or executive functions (*p*s > 0.091).

### Assessment of Executive Functions and Motor Functions and Order of Tasks

Three aspects of *executive functions* (motor inhibition, cognitive inhibition, and shifting; Miyake et al., 2000; Sereno et al., 2006) were assessed individually by means of two tasks at two times: at T1, 1 week before, and at T2, immediately after the coordinative intervention or control condition. Each task took approximately 10 min.

To assess *motor inhibition*, the “Simon-says” task (Strommen, 1973) was adapted from Carlson and Wang (2007). In this task, the children were asked to imitate ten simple movements, which had been named and performed first by the investigator who was facing the child (e.g., “touch your nose”). However, movements should only be imitated if the investigator said “Simon says” before naming and performing the movement (i.e., imitation trial). Otherwise, the child had to stay still and to suppress the imitation (i.e., inhibition trial). At the beginning of the “Simon-says” task, the investigator demonstrated all movements, which the child had to imitate, to ensure that he or she was able to perform these movements. Afterwards, practice trials were conducted as long as the child reacted correctly in an inhibition and a successive imitation trial. The following main task consisted of five imitation and five inhibition trials, which were presented mixed-up in one of two fixed orders. One second after the child imitated the movement – or after 3 s, if the child did not react – a new trial was demonstrated. After the fifth trial, the investigator reminded the child of the imitation rules. Only inhibition trials were considered in the statistical analyses. They were evaluated with a score between 0 and 3 (i.e., 0: full movement, 1: partial movement, 2: flinch, 3: no movement; Carlson and Meltzoff, 2008). Thus, across all five inhibition trials, a total score between 0 and 15 points could be achieved. The dependent variable was the percentage of the total motor inhibition score (i.e., accuracy in %).

*Cognitive inhibition* and *shifting* were assessed with the computer-based “Hearts-and-Flowers task” (Davidson et al., 2006) using E-Prime Software (Psychology Software Tools, Pittsburgh, PA, United States). The task was presented on a laptop (Dell, Vostro 3700, 17.3 inches, distance to monitor: 50 cm) and the child had to react to the trials on a separate keyboard that was placed in front of the child and which only consisted of a left and a right button. The child was presented with one of two stimuli: a heart or a flower, which had the same size (i.e., 3.8 cm × 3.9 cm) and color (i.e., red). The stimuli emerged on the right or the left side of a rectangle (7.2 cm × 28.6 cm), located in the center of the screen. There were three blocks (i.e., congruent, incongruent, and mixed), presented in a fixed order, each with 20 trials. In the first, *congruent block*, a heart appeared on the right or left side in the rectangle. The child had to press the button that was located on the same side as the heart. This block assessed the speed of information processing. In the second, *incongruent block*, a flower appeared on the right or left side in the rectangle. Now, the child had to push the button that was on the opposite side of the flower. Because of the dominant tendency to push the button on the same side on which the stimulus appears as the attention was focused to this side (i.e., Simon effect; Simon et al., 1976), the incongruent block required cognitive inhibition. In the third, *mixed block*, ten hearts and ten flowers appeared one after another in a fixed, pseudo-random order. The fixed order was chosen to realize the same difficulty (i.e., the same number of switches between congruent and incongruent trials) for all children and both times of measurement. Due to the permanent change of stimulus type (i.e., a total of 16 switches; the same stimulus type appeared maximally two times in succession), this block assessed shifting.

Children were instructed to react as quickly and accurately as possible in all blocks. Before the congruent and incongruent block started, children practiced the rules in at least four trials. Each practice trial was presented on the display until any button was pushed. The congruent and incongruent block started as soon as two of four successively shown practice trials were completed correctly. If the child reacted to less than two trials correctly, the four practice trials were repeated as long as two correct answers were given. Before the mixed block started, the two rules were repeated but no practice trials were executed. Each trial began with a fixation cross (500 ms), followed by a white slide (500 ms), the target stimulus (heart or flower, max. 1500 ms), and ended with another white slide (500 ms). The dependent variables were the mean reaction time (in ms) in the correct trials and the accuracy (in %) in the incongruent block, both assessing cognitive inhibition, and the mean reaction time (in ms) in the correct trials and the accuracy (in %) in the mixed block, both measuring shifting. We decided to not include the congruent block in the statistical analysis due to high accuracy rates and small variance between the children. All reaction times shorter than 200 ms were interpreted as random reactions and were excluded (Davidson et al., 2006). Furthermore, reaction times deviating more than three *SD* from the individual mean were also excluded (cf. Roebers and Kauer, 2009). Concerning accuracy, all trials were analyzed – independent of the exclusion of the associated reaction times – to treat random responses equally. Lacking reactions – no response within 2000 ms –were interpreted as wrong responses. Children with less than 20 % correct trials were excluded from the data analysis (*n* = 4).

The *order of the tasks* was counterbalanced between participants and was identical for both times of measurement within participants. There were two possible task orders: Simon says first or Hearts-and-Flowers task first. The three blocks of the Hearts and Flowers Task needed to be presented in sequential order to remain the same difficulty level for all children. Since it was assumed that the first task after the intervention could benefit the most, whereas the cognitive resources for the second task could be limited due to performing the first task, order of tasks was considered as an independent variable.

*Motor functions* were assessed with the German version of the “Movement Assessment Battery for Children – Second Edition” (M-ABC 2; Petermann, 2009). It consists of eight tasks that can be assigned to three scales: manual dexterity, ball skills, and balance. All children were examined with the task set for the age band 3–6 years. One child was already 7 years old, but as we did not use norm values and this child did not score at the maximum, this raised no problem. This test took about 20–30 min. For the correlative analyses, the raw scores of each task were *z*-standardized and summed up for each scale to realize a norm independent score. The sum of these three scores formed the total score for motor functions.

### Experimental Conditions: Coordinative Intervention and Control Condition

Each experimental condition took about 25 min (20 min exercise and 5 min instructions) and was executed with each child individually in the kindergarten. The order of the exercises in both conditions was counterbalanced between children. The acute *coordinative intervention* started with a 2 min running warm-up. Afterward, the children participated in four coordinative exercises (4.5 min each), which likewise required both sides of the body (bimanual and bipedal). The exercises included jumping in diverse combinations, balancing on a rope, bouncing a ball (Exercise 1), throwing balls on targets and running in diverse combinations (Exercise 2), kicking balls on targets and catching balls (Exercise 3), as well as boxing and kicking against a gymnastic ball (Exercise 4).

The acute coordinative intervention was adapted to the motor performance of each child during the intervention. Each exercise consisted of three to five difficulty levels with increasing motor and inhibition demands^2^. Whether a child achieved a higher level depended on the faults (e.g., missing a target) the child made on the previous level. For example, the second sub-exercise “throwing balls” with both hands consisted of three levels: On the first level, children should throw balls into a box within a distance of 1.5 m. If at least four of five balls were on target, the next level was reached, in which the box was placed in 2.0 m, and in the third level in 2.5 m distance from the child. Thus, only if a child made less faults, it could achieve a higher task level. A research assistant recorded the performance of the children during the intervention and signalized if the current exercise level was completed and if the child was allowed to proceed to a higher level.

The *control condition* also started with a warm-up (2 min.), in which the children stamped different pictures on freely chosen locations on a blank sheet of paper. Subsequently, four different tasks (4.5 min each) were executed: playing three different board games and watching a short movie. The board games included simple actions like pushing a button, putting objects in a container, or moving a meeple on the board in maximally three steps, depending on the number of points on a previously drawn card. The games were played interactively with short waiting times for the child to take the next action. Therefore, the execution of the tasks required little to no motor or cognitive resources.

### Manipulation Check

To measure the physical intensity in the coordinative intervention and control condition, children’s heart rate was assessed with a Polar RS800sd watch and a H1 sensor belt. According to the reversed U-shaped curve between physical arousal and cognitive performance (Yerkes and Dodson, 1908), a moderate physical intensity was expected to lead to optimal levels of cerebral blood flow and neurotransmitter release, and therewith to maximize cognitive performance (Timinkul et al., 2008; McMorris, 2009). Therefore, the aim was that the children in the coordinative intervention condition exercised on a moderate intensity level (i.e., 65–70% of maximal heart rate). The maximal heart rate (*HR*_max_) was estimated by the formula: *HR*_max_ *=* 208 – 0.7 *× age* (cf. Mahon et al., 2010). The children of the examined sample had a theoretical *HR*_max_ of approximately 204 beats per minute (bpm). Thus, a moderate intensity was reached by a target heart rate between 122 and 153 bpm. During the intervention, the heart rate was controlled every 20 s by the investigator and the frequency of movements was adapted to remain in the target heart rate range. A successful manipulation should yield a higher heart rate of the children in the coordinative intervention condition compared to children in the control condition. In fact, analyses revealed that children of the coordinative intervention exercised at a moderate intensity level (*M* = 136 bpm, *SD* = 9) that was significantly higher than in the control condition (*M* = 103 bpm, *SD* = 10), *t*(99) = 17.42, *p* < 0.001, *d* = 3.50.

Furthermore, we adapted the coordinative exercises to the motor functions of each individual child by means of applying different levels of task difficulty during the coordinative intervention (see Experimental Conditions: Coordinative Intervention and Control Condition). The descriptive statistics in **Table 1** confirm that children accomplished different levels in each coordinative exercise. In addition, the sum of the accomplished levels across coordinative exercises was positively correlated with total score of children’s motor functions, *r*(47) = 0.61, as well as with the sub-scores of manual dexterity, *r*(47) = 0.58, ball skills, *r*(49) = 0.37, and balance, *r*(49) = 0.51, *p*s < 0.001. This suggests that children with better motor skills completed the coordinative exercises on more advanced levels.

**Table 1:** Percentage of children who maximally reached a certain level in the intervention condition (*n* = 48), separately for each exercise.

Coordinative subtasks	None (%)	(1) Level (%)	(2) Level (%)	(3) Level (%)	(4) Level (%)	(5) Level (%)
Coordination ladder	0.0	8.3	16.7	18.8	43.8	12.5
Balance task	0.0	0.0	35.4	64.6	–	–
Bouncing task	10.4	31.3	37.5	20.8	–	–
Kicking goal task	2.1	79.2	18.8	0.0	–	–
Defending task	8.3	22.9	62.5	6.3	–	–
Throwing task						
Right hand	47.9	45.8	4.2	2.1	–	–
Left hand	64.6	27.1	8.3	0.0	–	–
Pasteboard task	0.0	8.3	12.5	60.4	14.6	4.2
Boxing task	2.1	10.4	56.3	31.3	–	–

*“–”: Task only contained three levels.*

### Statistical Analyses

Firstly, partial correlations between all executive and motor functions were calculated with age as control variable. Secondly, to check whether the dependent measures changed significantly from T1 to T2, we calculated paired *t*-tests with accuracy and mean reaction times of the three executive functions tasks as dependent variables. We also analyzed if there were any differences between the conditions concerning children’s motor and executive functions at T1. In addition, two mixed models^3^ were computed to examine if children in the coordinative intervention condition showed higher accuracies and lower reaction times at T2 in the executive function tasks than children of the control condition. In the mixed model with accuracy as dependent variable, planned comparisons were used to test whether this improvement was greater for motor inhibition compared to cognitive inhibition and shifting (task 1) as well as greater for cognitive inhibition than for shifting (task 2). Since reaction times were only recorded in the cognitive inhibition and shifting task, the mixed model with reaction times as dependent variable only allowed for a planned comparison between these two tasks. In addition, in both models the independent variables condition (coordinative intervention vs. control condition) and order of executive function tasks (Hearts-and-Flowers task first or Simon-says task first) were included. The independent variables could only be included as fixed effects as for random slopes a minimum of two observations is needed.

Each mixed model, analyzed with the package lmerTest (Kuznetsova et al., 2016) in the statistical computing software R (R Core Team, 2016), addressed that response data were nested within children and kindergartens and controlled for score differences at T1. Statistical assumptions (normal distribution and variance homogeneity) for the linear mixed models were visually checked by inspecting the residual plots and were judged as being sufficient.

## Results

Mean accuracy and reaction time of all three executive function tasks for both times of measurement as well as *z*-standardized scores of the motor functions for T1 are presented in **Table 2**, separately for each experimental condition. Two preliminary MANOVAs were calculated to check whether children of the coordinative intervention and the control condition differed at T1 concerning their performance in the motor function tasks and the executive function tasks. However, there was no difference between the two experimental conditions at T1, neither concerning children’s motor performance, *F*(3,95) = 0.30, *p* = 0.827, nor concerning their executive functions: accuracy: *F*(3,97) = 0.40, *p* = 0.396; reaction time: *F*(2,98) = 0.05, *p* = 0.954.

**Table 2:** Means and standard deviations of the executive functions and the *z*-standardized motor functions, separately for each experimental condition (*N* = 101).

	Condition			
	Control (*n* = 53)		Coordinative (*n* = 48)	
	T1	T2	T1	T2
**Executive functions**
Motor inhibition (ACC)	55.3 (39.8)	62.6 (41.0)	60.3 (35.9)	71.4 (33.7)
Cognitive inhibition (ACC)	76.6 (19.6)	86.1 (15.3)	78.8 (16.1)	90.4 (12.1)
Shifting (ACC)	55.2 (18.0)	67.3 (19.3)	53.1 (17.9)	67.4 (16.4)
Cognitive inhibition (RT)	782 (196)	695 (175)	793 (166)	705 (134)
Shifting (RT)	989 (223)	886 (194)	996 (167)	912 (191)
**Motor functions**				
Manual dexterity^2^	-0.01 (2.37)		0.05 (2.05)	
Ball skills	0.11 (1.69)		-0.12 (1.71)	
Dynamic balance	0.08 (2.14)		-0.04 (2.32)	
Total score^1,2^	0.15 (5.10)		-0.16 (4.83)	

*Standard deviation in parentheses; T1/T2, first/second time of measurement; ACC, accuracy in %; RT, reaction times in ms; ^1^Sum of *z*-standardized scores; ^2^*n* = 99, two children did not solve all tasks of manual dexterity.*

### Correlations between Motor and Executive Functions at T1

Partial correlations between motor and executive functions at T1, controlled for age, are presented in **Table 3**. In line with the first hypothesis, correlations between executive functions and motor functions at T1 were positive, ranging from small to moderate effect sizes (Cohen, 1988). Especially, the accuracy in the shifting task correlated moderately with all motor functions. In addition, the reaction times in the cognitive inhibition task correlated positively and moderately with all motor functions with exception of balance, whereas the accuracy in the cognitive inhibition task yielded no significant correlations to any motor function. Moreover, the reaction times of the shifting task were also not associated with any motor function. Motor inhibition only showed a significant positive moderate correlation to balance.

**Table 3:** Partial correlations between executive and motor functions across both experimental conditions at the pre-test T1, controlled for age (*N* = 101).

	Executive functions				
Motor functions^1^	Motor inhibition (ACC)	Cognitive inhibition (ACC)	Shifting (ACC)	Cognitive inhibition (RT)	Shifting (RT)
Manual dexterity^2^	0.25	0.09	0.38^∗∗^	-0.34^∗^	0.08
Ball skills	-0.01	0.11	0.36^∗∗^	-0.34^∗^	-0.07
Dynamic balance	0.33^∗^	0.26	0.39^∗∗^	-0.16	0.14
Total Score^2^	0.28	0.21	0.50^∗∗^	-0.35^∗^	0.08

*^1^Sum of *z*-standardized scores; ^2^*n* = 99, two children did not solve all tasks of manual dexterity; ^∗^*p* < 0.0025, ^∗∗^*p* < 0.0005; ACC, accuracy; RT, mean reaction time.*

### Effect of the Acute Coordinative Intervention on Performance in the Executive Function Tasks

#### Accuracy

**FIGURE 1:**
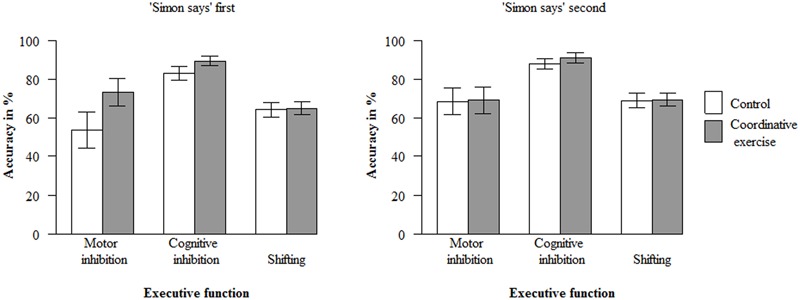
**Mean accuracy (in %) at T2, depending on condition, type of executive function task, and order of tasks.** Error bars indicate 95% confidence intervals.

Paired *t*-tests showed a significant gain in accuracy (in %) from T1 to T2 across the coordinative intervention and the control condition in motor inhibition, *t*(100) = -3.53, *p* < 0.001, *d* = -0.35, cognitive inhibition, *t*(100) = -6.19, *p* < 0.001, *d* = -0.62, and shifting, *t*(100) = -8.40, *p* < 0.001, *d* = -0.84. The mixed model with accuracy in the three executive function tasks at T2 as dependent variable yielded no significant main effect of the experimental condition, ß_1_ = 1.83, *t*(91.89) = 1.50, *p* = 0.137^4^. It should be noted that the power to detect differences between both conditions in the present sample, assuming a medium effect size of *f*^2^ = 0.15 (Cohen, 1988), was large enough: 1 – ß = 0.97 (Faul et al., 2007). Therefore, the second hypothesis had to be rejected: Given that there was no difference between children in the coordinative intervention condition and the control condition at T1 (see Preliminary analyses, 3.), the acute coordinative intervention did not lead to a higher overall gain of accuracy in the executive function tasks from T1 to T2 in contrast to the control condition. However, there was a significant three-way interaction between condition, type of executive function task (task1: motor inhibition vs. cognitive inhibition and shifting), and order of tasks, ß_11_ = 3.91, *t*(191.59) = 2.93, *p* = 0.004. *Post hoc* tests revealed that the accuracy in the motor inhibition task at T2 was higher in the coordinative intervention condition (*M* = 73.3%, *SD* = 44.5%) compared to the control condition (*M* = 53.9%, *SD* = 34.6%, ß = 9.77), *t*(39.33) = 2.87, *p* = 0.007, – but only if the Simon-says task was presented first (**Figure 1**)^5^. Thus, the coordinative intervention led to a larger improvement of motor inhibition than the control condition under a specific condition, which confirms at the same time our hypothesis that motor inhibition profits more (or at all) compared to other executive functions from a coordinative intervention. Furthermore, there was a significant effect of accuracy at T1 on the accuracy at T2 across all executive function tasks and conditions, ß_2_ = 0.66, *t*(256.53) = 16.16, *p* < 0.001: Children showed a higher accuracy in the executive function tasks at T2 if their accuracy was higher at T1. Moreover, the accuracy of the three executive function tasks (across both conditions) differed at T2: It was lower for motor inhibition compared to cognitive inhibition and shifting, ß_4_ = -3.95, *t*(195.27) = -2.92, *p* = 0.004, and lower for shifting compared to cognitive inhibition, ß_5_ = -2.78, *t*(213.34) = -2.23, *p* = 0.027. No other effects or interactions were significant.

#### Reaction Times

Paired *t*-tests showed a significant reduction of reaction times from T1 to T2 across both experimental conditions in cognitive inhibition, *t*(100) = 7.88, *p* < 0.001, *d* = 0.78, and shifting, *t*(100) = 5.48, *p* < 0.001, *d* = 0.55. Note that no reaction times were assessed in the motor inhibition task. The mixed model with mean reaction times (in ms) at T2 as dependent variable revealed no significant effect of the experimental condition, ß_1_ = 5.77, *t*(91.74) = 0.52, *p* = 0.608 (power corresponds to that of accuracy), and there were no significant interactions with this variable. Consequently, our hypothesis has to be rejected: Given that there was no difference between children in the coordinative intervention and the control condition at T1 (see Preliminary analyses, 3.), the coordinative intervention did not lead to a greater reduction of reaction times for the two executive functions in contrast to the control condition. However, there was a significant effect of reaction times at T1 on reaction times at T2 across both executive function tasks and conditions, ß_2_ = 0.55, *t*(193) = 11.32, *p* < 0.001: Children showed shorter reaction times at T2 if they had shorter reaction times at T1. In addition, across both conditions, children showed shorter reaction times at T2 in cognitive inhibition (*M* = 743 ms, *SD* = 175) than in shifting (*M* = 945 ms, *SD* = 200), ß_4_ = -42.37, *t*(132.3) = -5.12, *p* < 0.001^6^.

## Discussion

One aim of the present study was to examine the relationship between motor functions and executive functions in kindergartners. In particular, shifting (accuracy) and cognitive inhibition (mean reaction time) correlated significantly with almost every motor function whereas motor inhibition only showed a significant correlation with balance. A second aim was to investigate whether there is a causal effect of an acute coordinative intervention on different aspects of executive functions in kindergartners. In general, the acute coordinative intervention had no greater effect on executive functions of kindergartners than the control activity. However, if motor inhibition was tested as first executive function immediately after the intervention, the children of the coordinative intervention condition appeared to perform more accurately in the motor inhibition task than children of the control condition. These results are discussed in more detail in the following.

### Correlations between Motor and Executive Functions

Our findings concerning positive correlations between motor and executive functions are largely in line with previous research. For instance, motor functions were related to cognitive and motor inhibition (Livesey et al., 2006; Cameron et al., 2012), shifting (Mierau et al., 2016), and global measures of executive functions (Röthlisberger et al., 2010; Roebers et al., 2014) in kindergartners and older children. More specifically, manual dexterity, balance, and ball skills, used as indicators of children’s motor functions in the present study (cf. Petermann, 2009), were positively correlated with kindergartners’ executive functions. All of these motor functions require the precise execution and constant adaptation of movements, an elaborated coordination between visual perception and processes of motor movement. It can therefore be assumed that executive functions at least partly navigate these processes. Even though in the current study the effect sizes of several correlations between motor functions and motor inhibition on the one hand and accuracy in the cognitive inhibition task on the other hand were comparable to previous studies (e.g., Roebers and Kauer, 2009; Röthlisberger et al., 2010), they failed to reach significance when the significance level was adjusted for multiple testing. Furthermore, reaction times in the shifting task did not correlate with any motor function. Given children’s poor performance in this task (i.e., accuracy about 54%) and given the fact that only reaction times of correctly completed trials were considered, it can be presumed that mean reaction time in the shifting task is no reliable measure and therefore yielded no significant correlation with the motor functions.

To conclude, our correlational results suggest that motor functions and executive functions are interrelated in kindergartners. It can be assumed that these relationships base on shared developmental and learning mechanisms (e.g., Piaget, 1972; Ackerman, 1987; Best, 2010) as well as on the collective activation of an underlying neuronal network that connects brain regions being associated with motor and cognitive functions (Diamond, 2000). Furthermore, general biological maturation processes might lead to a parallel increase of executive and motor functions (Luo et al., 2007). The underlying causal mechanisms of these relationships are still not fully understood, but some studies indicated bi-directional effects between motor and executive functions (e.g., Weinert and Schneider, 1999; Roebers et al., 2014). One attempt to uncover a causal relationship between motor and executive functions was the implementation of an acute coordinative intervention.

### Effect of the Acute Coordinative Intervention on Executive Functions

In the current study, there was no general effect of the acute coordinative intervention on the examined executive functions in kindergartners. Children in both the intervention and control condition reacted faster and more accurately in the executive function tasks at T2 compared to T1. These results contradict studies reporting positive effects of coordinative interventions and aerobic exercise interventions on inhibition (Barenberg et al., 2011; Jäger et al., 2014) and shifting (Ellemberg and St-Louis-Deschênes, 2010; Chen et al., 2014), revealed for children older than 6 years, adolescents, and adults. However, other studies failed to find effects of acute aerobic exercise or coordinative interventions on shifting and inhibition of kindergartners (Mierau et al., 2014) as well as on shifting of adolescents (Kubesch et al., 2009) and of overweight children (Tomporowski et al., 2008a). One might assume that at least some of these contradicting findings could be assigned to differences in the general design of the mentioned studies (i.e., setting, in which the intervention was executed; type of acute physical activity; measures of executive functions; and examined age groups). If this is true, it also suggests that the causal effects of acute physical activity interventions on executive functions are not as robust as one might expect and emerge only under certain conditions that still need to be uncovered.

Besides expecting a general effect of the acute coordinative intervention, we assumed that the size of the effect would depend on the kind of executive function tested: The effect on motor inhibition should be greater compared to the effect on cognitive inhibition and shifting. Indeed, if motor inhibition was tested first, children of the coordinative intervention condition showed a higher accuracy in this task than children of the control condition. Even though this finding should be interpreted cautiously, it might be based on the closer correspondence between the coordinative intervention and the requirements of the motor inhibition task in contrast to the other executive function tasks: At higher difficulty levels of the coordinative intervention, the children had to inhibit whole-body movements based on specific rules and commands – an ability that is also required in the motor inhibition task. However, this effect did no longer emerge if motor inhibition was tested as second task after the intervention. One explanation could be that due to the cognitive demands of the first task, the cognitive resources to solve the second executive functions task were reduced. In the few studies with children older than 6 years of age and adolescents, examining more than one cognitive function, the positive effects of acute physical activity on some of these cognitive functions were reported independently of the order in which the tasks were presented (e.g., Cooper et al., 2012; Jäger et al., 2014). However, kindergartners’ attentional and cognitive resources are stronger limited than those of older children (Bjorklund and Harnishfeger, 1990; Best et al., 2009). Therefore, executing one cognitive task could reduce the cognitive resources for the following task. This assumption should be tested in future studies because so far, studies with kindergartners only examined the effect on one cognitive function at a time (e.g., Palmer et al., 2013; Mierau et al., 2014).

One reason for the lacking general effect of the coordinative intervention in the current study might be the setting in which the intervention took place. The interventions of the studies mentioned earlier, reporting positive effects, were often conducted either as *coordinative interventions in group setting*s (Chen et al., 2014; Jäger et al., 2014) or as *individually executed, aerobic exercises* (Hillman et al., 2009; Ellemberg and St-Louis-Deschênes, 2010; Pontifex et al., 2013). *Group settings* pose higher social demands as participants have to anticipate the intention and behavior of other participants, adapt their own behavior based on that and switch their behavior between changing conditions. This anticipation and adaption directly requires cognitive functions, such as attention and executive functions (Diamond, 2000), so that group settings could stimulate the pre-activation of these functions for a subsequent executive function task. The higher efficacy of acute interventions with a social component on neuronal brain structures was already shown for rats: Free wheel running in addition to living in groups led to a higher neurogenesis in the hippocampus (associated with memory) than individual wheel running of isolated living rats (Stranahan et al., 2006). Taken together, interventions in group settings might enhance cognitive functioning by their social demands and can have a high ecological validity in contrast to interventions in individual settings, but they also bear difficulties concerning the control of the physical intensity of the interventions and of the correct execution of movements.

*Aerobic exercise interventions* conducted in *individual settings* also found positive effects on diverse cognitive functions (cf. Hillman et al., 2009; Ellemberg and St-Louis-Deschênes, 2010; Pontifex et al., 2013). One advantage of those settings is that the physical intensity for each child can be individually adapted. Based on this adaption, a precise, moderate intensity level can be achieved, which provides an optimal arousal level for the subsequent cognitive performance (Yerkes and Dodson, 1908). Therefore, studies using an individual setting to implement physical activity have a high internal validity.

The intervention in the current study is a mixture of the designs mentioned above, including a *coordinative intervention* conducted in an *individual setting*. Even as it was a coordinative intervention, it was easier to control the physical intensity for each child, compared to a group setting. The only study with a partly similar design using an individual setting was conducted by Best (2012), testing the efficacy of one coordinative, cognitive engaging intervention and one intervention that only included repetitive movements in 6- to 10-year-olds. There was a positive effect for both interventions on inhibition. However, in contrast to the current study, the interventions were computer-based and therewith strictly controlled as each child received exactly the same procedure and executed the same amount of movements.

Taken together, it might be assumed that stable positive effects of acute interventions on executive functions can only be achieved if the interventions include social components (e.g., the interaction with others), or if they allow for controlling the physical intensity and the correct execution of movements (or both). More generally, it can be concluded that acute physical activity interventions can have positive effects on executive functions of kindergartners only under certain conditions. Thus, an acute intervention might not have a general, enhancing effect on executive functions.

An additional reason that might explain the temporally limited effect and the lack of a more general effect of the acute intervention on executive functions in the current study is the arousal level during the intervention, which could have been too low or rather inadequate. The physical intensity of the intervention was controlled to induce a moderate arousal, which should allow for an optimal cognitive performance (Yerkes and Dodson, 1908; McMorris, 2009). However, determining a moderate arousal for an individual child also depends on his or her aerobic capacity. The moderate intensity of the intervention in this study was only approximately estimated for all children, depending on their age, and not individually identified. Therefore, for some children the physical intensity could have been higher or lower than their individual moderate level, which could have resulted in a suboptimal arousal and, thus, an inadequate cognitive stimulation. Besides, the time children were actually physical active was interrupted four times for about 45 s to tell the instructions for the next exercise. These short pauses led to a decline in children’s heart rates, which could also have reduced the effectivity of the coordinative intervention.

The current study was the first to our knowledge that aimed to adapt the coordinative difficulty level of the exercises to the individual motor performance of kindergartners. The reason for this adaption was to stimulate the neuronal network between the cerebellum and the prefrontal cortex, and to achieve a pre-activation for the subsequent executive function tasks (cf. Diamond, 2000; Budde et al., 2008). One way to achieve this activation is by means of complex tasks (Diamond, 2000). The complexity of the coordinative tasks depends on the individual motor functions of the children. However, in the current study all children started at the same difficulty level which led to a longer exercise on a low difficulty level for children with high motor functions and therefore to a lower mean cognitive and motor demand in contrast to children with low motor functions. The adaptation thus was not optimal. In future studies, the individual performance during the intervention should be analyzed beforehand and participants should then start at different difficulty level depending on their motor performance.

Besides these points of concern regarding the acute physical activity intervention, a potential interaction between the complexity and the intensity of the intervention has not been taken into account. Pesce (2009) describes that the efficacy of an acute physical activity intervention on cognitive functions depends on the interaction between task-related characteristics (e.g., duration, intensity, complexity of the intervention and of the cognitive task) and individual characteristics (e.g., the individual level of aerobic capacity, coordinative, and cognitive abilities). These interactions have an influence on the cognitive resources that can be provided due to the physical activity (Pesce, 2009). For the interaction between intervention intensity and the complexity of the cognitive task, an inverted u-shaped curve was assumed (e.g., McMorris, 2009) and confirmed in several studies (e.g., Kamijo et al., 2007; Chang et al., 2011), whereby a moderate intensity level led to a moderate arousal and to an optimal performance in a complex cognitive task. However, it is still unknown how the interaction between the complexity of an acute physical activity intervention and the cognitive task influences the effect of the intervention as well as in which way the intensity level and the complexity interact. Some studies varied the complexity of the acute intervention, while keeping the intensity constant (e.g., Pesce et al., 2009; Best, 2012), with inconsistent results: Some studies found a greater effect for complex interventions (Budde et al., 2008; Pesce et al., 2009). Other studies showed comparable positive effects of interventions with high and low complexity on cognitive functions (Best, 2012; Jäger et al., 2015). In contrast, one study showed a detrimental effect for an intervention with high complexity, which was explained by a too high arousal level and therefore a suboptimal cognitive precondition (Gallotta et al., 2012). Similarly, in the current study the interaction between a moderate intensity and a moderate to high complexity level could have led to a mental or physical overload – in particular as here a younger sample was involved, compared to the above mentioned studies. Future studies should realize acute physical activity interventions with different demands concerning the complexity and intensity to allow for a more precise examination of the interaction between these interventional characteristics.

Another limitation of the current study derives from the measures used for assessing executive functions. To measure cognitive inhibition, the Hearts-and-Flowers task (Davidson et al., 2006) was applied although many studies assessed cognitive inhibition with the Flanker task (e.g., Eriksen and Eriksen, 1974; Chen et al., 2014; Jäger et al., 2015). It was chosen to avoid the potential ceiling effect concerning the accuracy of the Flanker task that was reported for kindergartners (e.g., Diamond et al., 2007). The Hearts-and-Flowers task therefore allows to measure change due to physical activity for a greater percentage of children. However, the tasks apparently assess different aspects of inhibition: While the Flanker task assesses the resistance against distraction, the Hearts-and-Flowers task measures the resistance against a predominant response. The Flanker task thus might be more sensitive to the effect of acute physical activity. This assumption is supported by Kubesch et al. (2009), who found a positive effect of 30 min aerobic exercise on the performance of adolescents in the Flanker task, but not in the Hearts-and-Flowers Task. An additional limitation of the applied measures is that only inhibition and shifting were assessed to represent the construct of executive functions without taking updating into account. In general, the evidence for the beneficial effects of acute physical activity on updating are inconsistent, including studies that found no effect (e.g., Cooper et al., 2012) and studies that found a positive effect (e.g., Jäger et al., 2015) for children and adolescents. Therefore, further studies should include measures for all three executive functions to allow for generalized predictions on the effect of acute physical activity on executive functions. Moreover, it has been suggested that executive functions do not only involve cognitive “cold” functions, as assessed in the present study, but also “hot” executive functions that refer to affective cognitive abilities (Zelazo and Müller, 2002). Such “hot” executive functions play a central role in many situations in which decisions have to be made that might have marked emotional consequences and that require the control of emotional arousal. Social and behavioral aspects of hot executive functions can be differentiated. Social aspects involve, for instance, negotiations with other persons or solving interpersonal conflicts. Behavioral aspects include abilities like waiting for a delayed gratification (Mischel et al., 1989) or choosing the less risky but less promising alternative in a gambling game (Kerr and Zelazo, 2004). “Hot” aspects of executive functions might also benefit from physical activity, especially if it is executed in group settings that involve emotionally relevant aspects, such as social comparisons or waiting until it is one’s turn. Thus, future research might widen the focus to uncover whether there are effects of physical activity on a broader range of executive functions.

Until now, little evidence exists for the positive effect of an acute intervention on cognitive functions of kindergartners. Palmer et al. (2013) showed that the attention of kindergartners benefitted from an acute coordinative bout, but there was only a marginal effect on inhibition. Similarly, the study of Mierau et al. (2014) and the current study did not find a general effect of an acute coordinative intervention on inhibition or shifting for this age group. Thus, the results from older children, adolescents, and adults could not be replicated for kindergartners so far. A possible explanation is the still poor maturation of the prefrontal cortex in kindergartners, a brain region that is associated with executive functions (Gogtay et al., 2004). Accordingly, the neuronal association between the prefrontal cortex and the cerebellum could not be developed sufficiently in order to enable a co-activation in complex executive function tasks or motor tasks. Therefore, basal cognitive functions like attention, which develops earlier (Garon et al., 2008), might be better abilities to be improved by an acute bout of physical activity in kindergartners than executive functions. Further studies with kindergartners are needed to draw a reliable conclusion if acute physical activity can benefit cognitive functions in this age group.

## Conclusion and Implications

Taken together, the current study revealed small to moderate relationships between executive and motor functions in kindergartners. Although the concrete underlying processes of this association are not fully understood, it could be assumed that motor and executive functions could affect each other bi-directionally. Nevertheless, in the current study the coordinative intervention did not lead to a larger gain of kindergartners’ executive functions in general, compared to a control condition. The intervention augmented only motor inhibition, if it was tested first after the intervention. Thus, there is no simple mode to enhance executive functions of kindergartners in general by acute coordinative interventions. Instead, such interventions might yield specific effects, depending on the design, and further research might uncover the conditions under which these effects occur. It might also be promising to investigate the effect of an acute bout of physical activity on more classroom learning related measures of executive functions that involve an emotional component (e.g., waiting for a turn or suppressing impulsive reactions) as well as effects on more basal cognitive functions in addition to executive functions in kindergartners. Moreover, it could be useful to measure neurophysiological (e.g., brain activity by means of event-related potentials) and physiological parameters (e.g., release of neurotransmitters) to analyse the underlying processes. Even if no overt effect of an acute bout of physical activity in behavioral measures is evident, compensatory mechanisms optimizing task performance could be uncovered by this means.

## Author Contributions

MS designed and conducted the study and wrote large parts of the manuscript. ME supported designing the study, wrote parts of the manuscript and critically revised it for important intellectual content. MA wrote large parts of the result section and supported the statistical analyses and interpretation of the data. All authors gave their final approval of the manuscript version to be published and agreed to be accountable for all aspects of the work in ensuring that questions related to the accuracy or integrity of any part of the work are appropriately investigated and resolved.

## Conflict of Interest Statement

The authors declare that the research was conducted in the absence of any commercial or financial relationships that could be construed as a potential conflict of interest.

## Footnotes

1 This study was carried out in accordance with the recommendation of the ethics committee of the Faculty of Human Sciences of the University of Kassel with written informed consent from all legal guardians of the subjects in accordance with the Declaration of Helsinki.

2 For an overview of each task, difficulty levels, and criteria to reach a higher level, see the Supplementary Material.

3

$$\begin{array}{c} ACC_{\text{ijk},\text{T}}{}_{2}\;=\;\beta_{0}\;+\;\beta_{1}\;condition_{\text{ijk}}\;+\;\beta_{2}\;ACC_{\text{ijk},\text{T}}{}_{1}\;+\;\beta_{3}\;order_{\text{ijk}}\;+\;\beta_{4}\;task1_{\text{ijk}}\;\\ \text{ +}\;\beta_{\text{5}}\;{\text{task2}}_{\text{ijk}}\;\text{+}\;\beta_{\text{6}}\;{\text{condition}}_{\text{ij}}{}_{\text{k}}\;{\text{order}}_{\text{ijk}}\;\text{+}\;\beta_{\text{7}}\;{\text{condition}}_{\text{ij}}{}_{\text{k}}\;{\text{task1}}_{\text{ijk}}\\ \text{ +}\;\beta_{\text{8}}\;{\text{condition}}_{\text{ij}}{}_{\text{k}}\;{\text{task2}}_{\text{ijk}}\;\text{+}\;\beta_{\text{9}}\;{\text{order}}_{\text{ij}}{}_{\text{k}}\;{\text{task1}}_{\text{ijk}}\;\text{+}\;\beta_{\text{10}}\;{\text{order}}_{\text{ij}}{}_{\text{k}}\;{\text{task2}}_{\text{ijk}}\\ \begin{array}{c} \text{ +}\;\beta_{\text{11}}\;{\text{condition}}_{\text{ijk}}\;{\text{order}}_{\text{ij}}{}_{\text{k}}\;{\text{task1}}_{\text{ijk}}\text{+}\;\beta_{\text{12}}\;{\text{condition}}_{\text{ijk}}\;{\text{order}}_{\text{ij}}{}_{\text{k}}\;{\text{task2}}_{\text{ijk}}\\ \text{+}\;{\text{u}}_{\text{0j}}\;\text{+u}{\;}_{\text{0k}}\text{+}\;\varepsilon_{\text{ijk}}\; \end{array} \end{array}$$

$$\begin{array}{c} {\text{RT}}_{\text{ijk},\text{T2}}\;\text{=}\;\beta_{\text{0}}\;\text{+}\;\beta_{\text{1}}\;{\text{condition}}_{\text{ijk}}\;\text{+}\;\beta_{\text{2}}\;{\text{RT}}_{\text{ijk},\text{T}}{}_{\text{1}}\;\text{+}\;\beta_{\text{3}}\;{\text{order}}_{\text{ijk}}\;\text{+}\;\beta_{\text{4}}\;{\text{task1}}_{\text{ijk}}\\ \text{ +}\beta_{\text{6}}\;{\text{condition}}_{\text{ij}}{}_{\text{k}}\;{\text{order}}_{\text{ijk}}\;\text{+}\;\beta_{\text{7}}\;{\text{condition}}_{\text{ij}}{}_{\text{k}}\;{\text{task1}}_{\text{ijk}}\\ \text{ +}\;\beta_{\text{9}}\;{\text{order}}_{\text{ij}}{}_{\text{k}}\;{\text{task1}}_{\text{ijk}}\;\text{+}\beta_{\text{11}}\;{\text{condition}}_{\text{ijk}}\;{\text{order}}_{\text{ij}}{}_{\text{k}}\;{\text{task1}}_{\text{ijk}}\\ \text{+}\;{\text{u}}_{\text{0j}}\;\text{+}\;{\text{u}}_{\text{0k}}\;\text{+}\;\varepsilon_{\text{ijk}}\; \end{array}$$

For each measurement *i*, child *j*, kindergarten *k*. *ACC* = accuracy (at T1 or T2), *RT* = reaction time (at T1 or T2), task1 = planned comparison for motor inhibition vs. cognitive inhibition and shifting, task2 = planned comparison for task cognitive inhibition vs. shifting, μ_0j_ random intercept for each child, μ_0k_ random intercept for each kindergarten.

4 All regression coefficients are unstandardized and all factors were effect coded with the exception of task (see Statistical Analyses).

5 The results were confirmed by a direct model comparison analysis, χ^2^(2) = 8.83, *p* = 0.012.

6 In addition, a bootstrap analysis confirmed the results of the linear mixed model.
