# Supplementary material for: Relationships between Motor and Executive Functions and the Effect of an Acute Coordinative Intervention on Executive Functions in Kindergartners
Source: Front Psychol. 2017 May 30;8:859. doi: 10.3389/fpsyg.2017.00859 (PMC5447760; doi:10.3389/fpsyg.2017.00859)
Supplement: Supplementary file 1 [file Table_1.docx]

Supplementary Material

Relationships between motor and executive functions and the effect of an acute coordinative intervention on executive functions in kindergartners

Marion Stein*, Max Auerswald, & Mirjam Ebersbach

*** Correspondence:** Corresponding Author: [Marion.Stein@uni-kassel.de](mailto:Marion.Stein@uni-kassel.de)

# Full description of the exercises in the acute coordinative intervention

Before starting the coordinative intervention, the following preparations were made. Every child got stickers on both hands and feet. The stickers on the child’s left hand and left foot were red, those on the right side were green. These stickers enabled the children to act according to the instructions of the experimenter who told the child therewith, which body side he or she should use. Afterwards, the warm-up phase began, in which the children had to run around a square (3.5 × 3.5 m). On every side of the square, the children had to imitate the running style of a different animal (i.e., bird, horse, crab, and octopus). On the side of the bird, the difficulty level was increased. Here, the children had to run through a coordination ladder (length: 3.5 m, distance between rungs: 36 cm) and were not allowed to touch the rungs of the ladder that was lying on the floor. After 2 min of warm-up, four coordinative exercises (à 4.5 min), each with increasing difficulty levels, were conducted in one of four different orders. In the following paragraphs, every exercise and its difficulty levels are explained.

## Exercise 1

In the first exercise, the child had to run or jump in diverse combinations through a coordination ladder (five difficulty levels), balance on a rope (three difficulty levels), run or jump through the coordination ladder again, and bounce a ball on targets on the floor (three difficulty levels). The different *jumping combinations* used with the coordination ladder, increasing in difficulty, are listed in Table S1. For each run through the ladder, a maximum of two faults (i.e., touching the ladder or using the wrong foot) were permitted to proceed to the next level. At the first difficulty level of the *balance task*, the child had to balance on a 1cm wide rope without losing touch with it (first difficulty level). At the second level, the child additionally held one ball in both hands, and at the third level, he or she held two balls while balancing. A maximum of two faults (i.e., stepping beside the rope, losing the ball/s) was allowed to reach the next level. For the *bouncing task*, six crosses (alternately one red and one green cross) were stuck on the floor in a line. At the first difficulty level, the child had to bounce the ball with both hands on each cross. At the second difficulty level, the child had to bounce the ball with the red hand on the red crosses and with the green hand on the green crosses. At the last difficulty level, the child had to use the hand with the opposite color of the cross to bounce the ball. At all difficulty levels, the child had to catch the ball after bouncing it onto each cross. A maximum of two faults (i.e., using the wrong hand, no catching) were permitted to proceed to the next level.

## Exercise 2

In the second exercise, the child had to kick or “defend” balls (each subtask with three difficulty levels). In the *kicking subtask*, the child kicked a green and a red ball (ø 17 cm) in a goal (width: 3 m) from 4 m distance. Afterwards, the child had to run 1.5 m to the right and 1.5 m to the left of the kicking start point to maintain a moderate intensity level. After ten scored goals in the kicking task, the child proceeded to a higher difficulty level. At the first difficulty level, the goal was scored if the child kicked the green ball with the green (right) foot and the red ball with the red (left) foot and hit the goal. On the next level, the colors were switched: Now, the green ball had to be kicked with the red foot and the red ball with the green foot, and again the goal had to be hit. The last level contained kicking the ball backwards with the heels according to the primary color attribution. In the *defending balls* *subtask*, the child had to defend a small soft ball (ø 8 cm) that was thrown to him or her by the experimenter (distance: 2 m) in different ways each time after running to the left and right side. At the first difficulty level, the child had to catch the ball with both hands. At the second difficulty level, the child should touch the ball with the head (i.e., doing a header). At the last level, the experimenter told the child shortly before throwing the ball which hand he or she should use by naming the correspondent color. The child had to defend the ball with the appropriate hand. In this subtask, after four correctly defended balls the children were allowed to proceed to the next level.

## Exercise 3

In this exercise, a *throwing subtask* and a *pasteboard subtask* alternated. In the *throwing subtask*, the child threw five soft balls (ø 8 cm) successively with the green hand in a 1.5 m distant box (57 × 39 × 28 cm). It included three difficulty levels depending on the distance to the box: (a) 1.5 m, (b) 2 m and (c) 2.5 m. If the child hit the box four out of five times with the correct hand, he or she went on to the next level for the specific hand. After throwing five times, the *pasteboard subtask* followed. Here, the child had to run in diverse movement patterns on red and green pasteboards (ca. 50 pieces) to the opposite side. The pasteboard subtask consisted of five difficulty levels: (1) run on the boards without touching the ground, (2) run only on the red boards, (3) run only on the green boards, (4) run with the red foot on the red boards and with the green foot on the green boards and (5) color switch (green foot on the red boards and vice versa). Reaching the other side, the child had to throw five soft balls with the red hand in the box (see *throwing subtask* above). Only one fault was allowed in each subtask to proceed to the next difficulty level.

## Exercise 4

This task included *kicking and boxing* against a gymnastic ball (ø 65 cm) held by the experimenter. If the ball was hold on the height of the child’s head (high), the child had to box against it. If it was hold knee-high (low), the child had to kick the ball. In addition, the experimenter named the color (= side of the body) with which the ball should be kicked or boxed. After every command, the experimenter and the child made two side steps to the left or right to maintain a moderate intensity level. The order of all commands is listed in Table S2. If the child made less than four faults during one cycle (consisting of 12 commands), the next level was reached. At the first level, the child only had to react to the height of the ball (box or kick) and the color command of the experimenter (green or red). The next level additionally contained that the child had to duck down if the ball was swung around the experimenter’s axis. This happened after every fourth command. In addition, at the third level the experimenter bounced the ball on the ground after every third command, which was the signal for the child to jump as high as he or she could. At the last difficulty level, a color switch was added: If the experimenter now named the color red, the child had to use the green side and vice versa.

Table S1

*Run and jump combinations for the coordination ladder subtask*

| Level | Combinations | Visual illustration |
| --- | --- | --- |
| 1) | Normal running (without touching the rungs) | 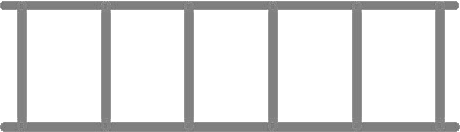 |
| 2) | Jumping with both feet simultaneously | 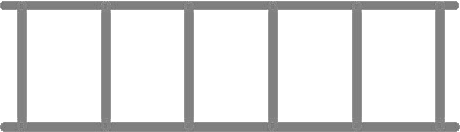 |
| 3) | Green foot – both feet – red foot | 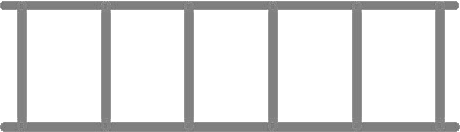 |
| 4) | Red foot – green foot – both feet | 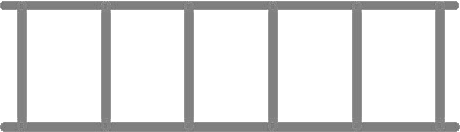 |
| 5) | Both feet – green foot (outside of the ladder) – both feet – red foot (outside) – both feet | 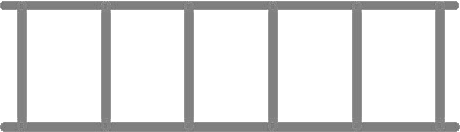 |

Table S2

*Commands of the third difficulty level of the kicking and boxing task, called out by the experimenter*

|  | **Position and color commands (meaning)** | **Additional cues (and associated actions)** |
| --- | --- | --- |
| 1 | High, green (box, right) |  |
| 2 | Down, red (kick, left) |  |
| 3 | Down, green (kick, right) | Bouncing the ball (jump high) |
| 4 | High, red (box, left) | Swinging the ball (duck down) |
| 5 | Down, green (kick, right) |  |
| 6 | High, green (box, right) | Bouncing the ball (jump high) |
| 7 | Down, red (kick, left) |  |
| 8 | High, red (box, left) | Swinging the ball (duck down) |
| 9 | Down, green (kick, right) | Bouncing the ball (jump high) |
| 10 | High, red (box, left) |  |
| 11 | High, green (box, right) |  |
| 12 | Down, red (kick, left) | Bouncing the ball (jump high)  Swinging the ball (duck down) |
